# Advances in Radiotherapy for Glioblastoma

**DOI:** 10.3389/fneur.2017.00748

**Published:** 2018-01-15

**Authors:** Justin Mann, Rohan Ramakrishna, Rajiv Magge, A. Gabriella Wernicke

**Affiliations:** ^1^Department of Radiation Oncology, Weill Cornell Medical College, New York, NY, United States; ^2^Department of Neurological Surgery, Weill Cornell Medical College, New York, NY, United States; ^3^Department of Neurology, Weill Cornell Medical College, New York, NY, United States

**Keywords:** glioblastoma, external beam radiotherapy, salvage radiotherapy, immunotherapy and radiotherapy, elderly fractionation, subventricular zone, hypofractionation, elderly

## Abstract

External beam radiotherapy (RT) has long played a crucial role in the treatment of glioblastoma. Over the past several decades, significant advances in RT treatment and image-guidance technology have led to enormous improvements in the ability to optimize definitive and salvage treatments. This review highlights several of the latest developments and controversies related to RT, including the treatment of elderly patients, who continue to be a fragile and vulnerable population; potential salvage options for recurrent disease including reirradiation with chemotherapy; the latest imaging techniques allowing for more accurate and precise delineation of treatment volumes to maximize the therapeutic ratio of conformal RT; the ongoing preclinical and clinical data regarding the combination of immunotherapy with RT; and the increasing evidence of cancer stem-cell niches in the subventricular zone which may provide a potential target for local therapies. Finally, continued development on many fronts have allowed for modestly improved outcomes while at the same time limiting toxicity.

## Introduction

Glioblastoma (GBM) is the most common primary brain tumor in adults and often occurs in patients over 65 years of age (1). Historically, the treatment for GBM had consisted of maximal safe resection followed by an adjuvant nitrosurea, with trials by the Brain Tumor Study Group demonstrating evidence for post-op RT over best supportive care (2, 3). Further analysis of the relationship between survival and radiation dose revealed a dose–effect relationship, with doses of 60 Gy providing superior survival when compared to lower doses (4). In 2004, a prospective, randomized, phase 3 trial by the European Organization for Research and Treatment of Cancer (EORTC) 26981-22981/National Cancer Institute of Canada Clinical Trials Group (NCIC CTG) reported improved progression-free and overall survival for patients with GBM with the addition of concomitant and adjuvant temozolomide (TMZ), an oral alkylating agent, to radiotherapy (RT) following maximal safe resection (5). The study was limited to patients with a World Health Organization (WHO) performance status of 2 or less and patients 18 to 70 years of age. The median progression-free survival was 6.9 (5.8–8.2) months in the TMZ arm vs 5.0 months with RT alone (*p* < 0.001). Median survival in the RT with TMZ arm was 14.6 vs 12.1 months in the RT alone arm, indicating the superiority of adding temozolamide to standard RT. Two more recent trials evaluated the treatment of newly diagnosed GBM with RT and both concurrent TMZ and bevacizumab, but showed no overall survival benefit (6, 7). While these trials were not meant to assess the effect of RT, its inclusion ofcourse indicates wide spread acceptance of RT as an important adjunct in the management of GBM. These results provide further evidence that surgery followed by RT with concurrent and adjuvant TMZ represents the standard of care for newly diagnosed GBM.

Though radiation is considered part of the standard of care for the treatment of GBM, there remain many areas of controversy and innovation. These include safe radiation regimens for the elderly or frail, reirradiation options in previously treated patients, imaging techniques to improve safety and efficacy, the potential synergy of immunotherapy and radiation, and finally the possibility of stem cell-directed radiation therapies aimed at reducing recurrence. In this review, we highlight these areas with an eye toward future developments in radiation therapy as it applies to GBM.

## Elderly Fractionation Regimens

There are no standardized guidelines for the treatment of GBM in elderly patients, likely related to their exclusion from large randomized trials (8). The most significant prognostic factor in GBM is age, followed by performance status, and molecular characteristics (9). In some instances, this has lead to undeserved treatment nihilism as it pertains to treatment of the elderly with GBM. A retrospective review of 206 GBM patients >70 years of age showed that higher Karnofsky Performance Status (KPS) and each treatment modality (surgery, RT, and chemotherapy) have a positive independent effect on improving survival, with patients having a KPS of 90–100 having a median survival of 17.2 months while those with a KPS of 80 had a median survival of 7.3 months (10, 11). The use of RT has been demonstrated to improve overall survival in elderly patients with GBM vs those receiving best supportive care, without significantly affecting cognition or quality of life. A phase 3 trial evaluated patients over the age of 70 with GBM or anaplastic astrocytoma (AA) with a KPS of 70 or more, who after surgery were randomized to either supportive care or focal RT, which was 50.4 Gy given in daily fractions of 1.8 Gy. Median survival was 29.1 vs 16.9 weeks and the hazard ratio for death in the RT group was 0.47 [95% confidence interval (CI), 0.29 to 0.76; *p* = 0.002] showing a significant survival benefit in those patients receiving adjuvant RT (12).

Given the generally worse prognosis of the elderly with GBM, additional studies have assessed the role of shorter courses of RT and whether they show equivalence to standard fractionation (6 weeks of radiation, given in daily fractions of 1.8 Gy). Shorter courses of RT, also known as hypofractionation, entail giving a larger daily dose (>1.8 Gy per fraction), which leads to a shorter duration of overall treatment. The first randomized trial appraising the effectiveness of an abbreviated RT course for elderly patients showed no difference in survival between 6-week and 3-week courses of RT (13). The Canadian trial enrolled patients ≥60 years old with a KPS of at least 50 and randomized them to standard adjuvant RT (60 Gy in 30 fractions over 6 weeks) or hypofractionated RT (40 Gy in 15 fractions over 3 weeks). The median survival was 5.1 months for the standard course vs 5.6 months for the hypofractionated course (NSS). The study’s secondary endpoint of health-related quality of life showed no significant difference between arms. Additionally, fewer patients in the hypofractionated arm required an increase in dosage of posttreatment corticosteroids. The study authors concluded that these results showed no significant difference in overall survival, 6-month survival, or quality of life between standard RT and a 3-week hypofractionated course, indicating that hypofractionated RT is a reasonable treatment option for elderly patients with GBM.

The median survival for elderly patients with GBM remains approximately 8 months with RT alone (14). Because the tolerability of combination RT and TMZ in the elderly seems to be reduced, the German Neuro-oncology Working Group (NOA) completed a randomized phase 3 trial (NOA-08) assessing whether dose-dense TMZ alone could substitute for standard fractionation in patients older than 65 years with a KPS of 60 or more (15). This study included 412 randomized patients following resection or biopsy to TMZ alone or RT alone. TMZ was 100 mg/m^2^ daily given on an alternating 1-week on, 1-week off schedule. RT was conventional fractionation of 60 Gy (1.8–2.0 Gy per fraction) delivered over 6 weeks. Median overall survival was 8.6 months in the TMZ group vs 9.6 months in the RT group, however as per the study protocol, TMZ was non-inferior to RT. O6-methylguanine-methyltransferase (MGMT) promoter methylation was analyzed for a subgroup of 209 patients, and the event-free survival (EFS) was longer in patients who received TMZ (8.4 vs 4.6 months), whereas the EFS was longer in patients without MGMT promoter methylation who received RT (4.6 vs 3.3 months). These results supported the use of TMZ alone as an alternative to RT alone in certain elderly patients; however, significant toxicity was seen in the TMZ alone arm, thus a conventional TMZ schedule may be preferable in elderly patients, especially since Radiation Therapy Oncology Group (RTOG) 05-25 trial failed to demonstrate improved efficacy of dose-dense TMZ when compared to standard TMZ (16). Furthermore, both NOA-08 and RTOG 05-25 confirmed the prognostic significance of MGMT promoter methylation status, which was strongly predictive for outcomes. The Nordic trial evaluated elderly GBM patients ≥60 years old by randomizing patients following resection between adjuvant treatment with TMZ alone (200 mg/m^2^ on days 1–5 every 28 days for up to six cycles), hypofractionated RT (34 Gy in 10 fractions given over 2 weeks), or standard RT given over 6 weeks (17). A total of 291 patients were randomized into one of the three arms and the primary endpoint was overall survival. When compared with standard RT, there was a statistically significant increase in median overall survival with TMZ (8.3 vs 6.0 months, *p* = 0.01), but not with hypofractionated RT (7.5 months, *p* = 0.24). However, for patients who received TMZ or hypofractionated RT, overall survival was not significantly different (8.4 vs 7.4 months, *p* = 0.12). Limiting analysis to patients over 70 years of age demonstrated better survival in the TMZ alone arm and the hypofractionated RT arm, compared to the standard RT arm (HR for TMZ vs standard RT 0.35, *p* < 0.0001; HR for hypofractionated vs standard RT 0.59, *p* = 0.02). Similar to the German trial, patients with MGMT promoter methylation who were treated with TMZ had better survival than those without (9.7 vs 6.8 months, *p* = 0.02); however, MGMT promoter methylation status did not affect survival in patients treated with RT. The authors concluded that for elderly patients (>70 years), standard RT is supplanted by either TMZ or hypofractionated RT, and MGMT promoter methylation status may be useful for predicting benefit from TMZ.

In 2015, the International Atomic Energy Agency published results from a randomized phase 3 trial of RT in elderly or frail patients randomized to two regimens of hypofractionated RT: 40 Gy in 15 fractions over 3 weeks vs 25 Gy in 5 fractions over 1 week (18). The definition of frail included patients ≥50 years and a KPS of 50–70, while elderly was defined as age ≥65 years with a KPS >70. The 1-week long course of RT was non-inferior to the 3-week course, with a median overall survival time of 7.9 vs 6.4 months (*p* = 0.716), thus failing to reject the null hypothesis of a difference between the two fractionations. Despite this significantly condensed short of treatment, there were no grade ≥3 acute toxicity and mean doses of corticosteroids did not differ in either arm at baseline or 1 or 3 months following treatment. This evidence showing no detriment to a 1-week course of RT provides the ability to treat this patient population in a manner that improves the survival-to-treatment time ratio, as the authors argue, and demonstrates striking advantage when cost–utility analysis are performed.

The NCIC CTG CE.6/EORTC 26062-22061/Trans-Tasman Radiation Oncology Group 08.02 elderly GBM study was a prospective, phase 3 trial that evaluated the benefit of hypofractionated RT given as 40 Gy in 15 fractions over 3 weeks with or without concomitant temozolamide and adjuvant temozolamide. This trial was limited to patients ≥65 years with good performance status (ECOG 0-2) randomized to hypofractionated RT (40 Gy in 15 fractions) with or without 3 weeks of concomitant TMZ plus monthly adjuvant TMZ until progression or 12 cycles. The final results showed that RT with concomitant and adjuvant TMZ significantly improved median overall survival (9.3 vs 7.6 months, *p* < 0.0001) and median progression-free survival (5.3 vs 3.9 months, *p* < 0.0001) over RT alone. The overall survival was almost double for patients with MGMT promoter methylation who received RT and TMZ compared to RT alone (13.5 vs 7.7 months, *p* = 0.0001). There was a trend toward improved survival for patients with unmethylated MGMT who received RT + TMZ over RT alone (10.0 vs 7.9 months, *p* = 0.055). Quality of life analysis showed no difference in physical, cognitive, emotional, or social functioning between the two groups. The experimental arm receiving concurrent and adjuvant TMZ had increased nausea, vomiting, and constipation, as would be expected. The addition of TMZ to hypofractionated RT was well tolerated with over 97% of patients completing the 3-week regimen of chemoradiation (14). Based on these results, the addition of concomitant and adjuvant TMZ to a 3-week course of RT should be considered for elderly patients with GBM.

These data provide further evidence supporting the use of concomitant chemoradiation for highly functioning elderly patients. For elderly patients with normal to above-normal performance status following resection, TMZ with either standard fractionation or hypofractionated RT (40 Gy in 15 fractions over 3 weeks) are reasonable treatment options, as there is no evidence that conventionally fractionated RT (60 Gy in 30 fractions) is superior to hypofractionated RT (19). For elderly patients with a KPS <70, then single-modality management may be reasonable, including TMZ alone for patients with an MGMT promoter methylated tumor, whereas patients with an unmethylated MGMT promoter may benefit from RT alone, for which the treatment plan should be individualized and given in a hypofractionated course over 1–3 weeks.

## Salvage RT

Salvage options for GBM are crucial given that a majority of patients will develop a recurrence following standard of care surgery, RT, and TMZ (5). Treatment options at time of recurrence include supportive care, reoperation, repeat RT (reirradiation), systemic therapy, or combined-modality therapy. The main determinants of prognosis are a favorable performance status (KPS ≥70) and younger age, which correlate with improved outcomes following salvage therapy (20). Other less strong prognostic factors include smaller tumors, non-eloquent brain location, greater interval from initial treatment to recurrence, and corticosteroid dependence.

Salvage reirradiation has been studied in multiple settings, including GBM. There are numerous published studies looking at reirradiation, mostly retrospective, utilizing a variety of treatment techniques, including 3D conventional RT, intensity-modulated radiotherapy (IMRT), brachytherapy, stereotactic fractionated RT, and stereotactic radiosurgery (SRS) with or without systemic therapy. Improvements in RT and imaging technology have allowed for more accurate delineation of treatment volumes and increased conformality of treatment, limiting toxicity to adjacent normal tissue. IMRT allows for a further increase in conformality by using a number of modulated beams of different intensities aimed at many different angles. SRS provides for even more precise targeting of treatment volumes while sparing surrounding critical structures, utilizing multiple beam sources with a steep dose gradient at the edge of target. Stereotactic treatment is usually limited to smaller sized treatment volumes, as the risk of toxicity increases with higher irradiated volumes. Patients may be immobilized with a frame-based or frameless technique, depending on the treatment machine (Cyberknife^®^, Novalis^®^, Gamma Knife^®^, etc.) Fractionated stereotactic RT is similar to radiosurgery, except that treatment can be divided over several days into multiple fractions, as it usually involves frameless immobilization. On board image guidance is used prior to and sometimes during each fraction, to ensure reproducibility of patient positioning. Fractionated stereotactic radiation has the innate radiobiological advantage of normal tissue repair between fractions, allowing for safer treatment of larger volumes while respecting normal tissue tolerances.

Bevacizumab has also been studied as an adjuvant to salvage irradiation. In 2009, the Food and Drug Administration approved bevacizumab for recurrent GBM. Bevacizumab is a humanized monoclonal antibody against vascular endothelial growth factor A, which decreases microvascular growth. This approval was based on significant response rates seen in various phase 2 studies and a meta-analysis by Wong et al. evaluating 15 studies published from 2005 to 2009, involving over 500 patients treated with bevacizumab for recurrent GBM. This meta-analysis showed a median overall survival of 9.3 months (95% CI, 7.9–10.6 months) and 6-month progression-free survival of 45% (95% CI, 34–57%) (21). Multiple phase 2 studies have tested the combination of bevacizumab with other chemotherapeutic agents, including carboplatin, irinotecan, TMZ, etoposide, erlotinib, and nitrosureas, which have all shown increased toxicity with no substantial improvement in efficacy, when compared to single-agent bevacizumab (22–26). Salvage chemotherapy following bevacizumab failure has shown limited success, with a retrospective study showing a combination of bevacizumab with another chemotherapeutic agent having a 6-month progression-free survival of 2% on the second regimen, indicating that patients who progress on a bevacizumab-containing regimen rarely respond to a second bevacizumab-containing chemotherapy combination (27).

A review by Nieder et al. included data from over 300 GBM patients receiving various forms of reirradiation as a salvage treatment modality including external beam RT, fractionated stereotactic RT, radiosurgery, brachytherapy, and combined RT and thermotherapy. Cumulative results yielded a 6-month progression-free survival of 28–39% and 1-year overall survival of 18–48%. Patients with KPS < 70 seemed to derive less benefit from treatment, although clinical improvement was seen in 24–45% of patients; therefore, stabilization of performance status was believed to be a realistic aim of treatment (28).

A retrospective study by Combs et al. described results of 147 recurrent gliomas (59 patients with GBM), who were treated with fractionated stereotactic RT, receiving a median dose of 36 Gy in 2-Gy fractions (29). Progression-free survival for GBM patients following reirradiation was 5 months, with a median overall survival after reirradiation of 8 months. Treatment was well tolerated with only a single case of radionecrosis. Factors significantly associated with improved survival included tumor histology (with lower grade gliomas portending better prognosis), extent of initial resection, and age at primary diagnosis.

A large retrospective study of reirradiation by Fogh et al. analyzed 147 patients with high-grade glioma treated with fractionated stereotactic RT (median dose, 35 Gy in 10 fractions) showed a median survival time of 11 months from salvage treatment, independent of reoperation or concomitant chemotherapy (30). Excellent responses were seen in patients who had recurred within 6 months of initial therapy, with non-inferior survival to those who recurred later than 6 months. Treatment was well tolerated with no significant acute morbidity from treatment, no treatment breaks or hospitalizations. On multivariate analysis, younger age and small treatment volume was associated with improved overall survival. Additionally, a study by Vordermark et al. evaluated the use of fractionated stereotactic RT for previously irradiated patients with recurrent GBM and AA receiving a median total dose of 30 Gy (median 5 Gy/fraction). For patients with GBM, the median overall survival was 7.9 months from time of reirradiation (31). The median time to radiographic progression after reirradiation was 4.9 months. Treatment was well tolerated with no patients requiring reoperation for symptomatic radiation necrosis.

There have also been multiple studies looking at the use of SRS in the salvage setting, usually treating with a single high-dose fraction of radiation. RTOG 90-05 was a phase 1 dose-escalation study that demonstrated SRS could be safely performed in patients who previously received RT for primary brain tumors and brain metastases, with acceptable morbidity (32). The maximum doses of single fraction radiosurgery defined as 24, 18, and 15 Gy for tumors ≤20, 21–30, and 31–40 mm in maximum diameter, respectively, with increasing risk of CNS toxicity, including radionecrosis, with increasing treatment volumes, thus radiosurgery is an option for limited volume disease recurrences. Multiple retrospective studies have published their outcomes from utilizing SRS monotherapy as salvage treatment for previously irradiated GBM, with median doses ranging from 13 to 20 Gy in a single fraction and median survival ranging from 8 to 11 months from time of radiosurgery (33–36). Regarding efficacy, the tumor dose was not predictive of outcome; however, younger age, higher KPS, and smaller treatment volumes were associated with improved survival. SRS was also shown to have a higher rate of radiation necrosis vs fractionated SRS, with one study showing 30% of patients receiving SRS developing radiation necrosis (35).

In the studies above, a large number of patients experienced significant improvement or resolution of neurologic symptoms and reduction of corticosteroid dose, heralding an improvement in functional status at 6 months. Given the low toxicity of focal reirradiation and evidence supporting improved outcomes compared to supportive care or systemic therapy alone, the clinical practice guidelines approved by the American Society of Radiation Oncology (ASTRO) endorses salvage reirradiation for recurrent GBM following completion of standard first-line therapy for younger patients with good performance status and limited tumor recurrence; however, there is not yet enough data to establish the optimal dose and technique (19). With the aforementioned evidence supporting the use of bevacizumab or reirradiation alone as salvage treatment, later studies have proceeded to evaluate the combination of reirradiation with concurrent and adjuvant bevacizumab.

The rationale of combining radiation and bevacizumab is based on the finding that bevacizumab works through multiple mechanisms as a radiosensitizer. Preclinical data show that ionizing radiation leads to upregulation of VEGF, which could be blocked by antiangiogenic therapies (37). Combination of RT and bevacizumab may lead to normalization of tumor vasculature, increasing radiosensitivity through a decrease in hypoxia, inhibition of repopulation following radiation, and inhibition of radiation-induced upregulated VEGF expression (38). Additionally, bevacizumab is believed to have radioprotective effects on normal tissue through vascular normalization and reduction of vascular permeability, which may reduce the likelihood of brain radionecrosis following reirradiation and even show benefit when used for the acute treatment of symptomatic radiation necrosis (39).

In the first pilot study of its kind combining reirradiation and bevacizumab, Gutin et al. treated 25 patients (20 of which had GBM) with a median age of 56 years (range, 30–80) and median KPS of 90 (range, 70–100) with salvage reirradiation with concurrent and adjuvant bevacizumab (40). All patients received bevacizumab (10 mg/kg IV) every 2 weeks of a 28-day cycle until failure and received fractionated stereotactic RT (30 Gy in five fractions) 5 days after the first cycle of bevacizumab. Among GBM patients, overall response rate was 50% with a 6-month progression-free survival of 65%. Median overall survival was 12.5 months and 1-year survival was 54%. Three patients on the study discontinued treatment due to toxicity, including one patient with a grade 3 CNS intratumoral hemorrhage, one with a bowel perforation in the setting of chronic steroid use, and one patient who developed craniotomy wound dehiscence requiring repair. The latter patient had the initial craniotomy 4 weeks prior to starting bevacizumab. A fourth patient developed a lower gastrointestinal bleeder 3 weeks after coming off study. Importantly, no patients experienced clinical or radiographic radiation necrosis. The authors contend that these complications were comparable to other reports of bevacizumab use in patients with GBM. The combination of bevacizumab and RT was thought to have an improved therapeutic ratio as there were no cases of radionecrosis and no increased need for corticosteroids during or after RT. The results of this study compare favorably to the many chemotherapy studies previously discussed and showed that the combination of bevacizumab and fractionated stereotactic RT was safe and tolerated well (40). The fractionation scheme of 30 Gy in five fractions (6 Gy/fraction) was modeled on the Vordermark et al. study described previously (31). Another retrospective single-institution study of high-grade glioma patients treated with salvage fractionated stereotactic RT (36 Gy in 18 fractions) with or without concurrent and adjuvant bevacizumab showed improved overall survival in the combination arm with the mean survival of 187.4 days after reirradiation alone compared to 367.6 days after reirradiation plus bevacizumab, thus demonstrating that there is sufficient statistical evidence to suggest that the survival for patients treated with and without combination bevacizumab differ (41).

The ongoing RTOG 12-05 randomized phase 2 trial is evaluating bevacizumab with or without RT in patients with recurrent GBM. This study should provide further understanding regarding whether reirradiation with bevacizumab is beneficial compared to bevacizumab alone. Additionally, other trials are evaluating the combination of immunotherapy with reirradiation and bevacizumab.

## Novel Treatment Planning

When designing a RT plan for GBM, conventional treatment volumes are based on an initial gross tumor volume (GTV) based upon a postoperative-enhanced MRI with the goal of delineating the surgical resection cavity plus any residual enhancing tumor. This initial GTV is given an additional clinical target volume (CTV) expansion, or margin, for which there remains significant variation in size. The EORTC currently recommends a 2- to 3-cm CTV margin and a 3- to 5-mm planning target volume (PTV) expansion, which is meant to account for any inaccuracies and uncertainties of treatment planning to ensure that the prescribed dose is actually delivered. This entire volume is prescribed at a conventional dose of 60 Gy in 2 Gy daily fractions (42). Alternatively, the RTOG uses a larger initial volume, based on the T2 or FLAIR abnormality on the postoperative MRI with a 2 cm CTV margin and 3–5 mm PTV margin followed by a “conedown” (also called a “boost”) phase with a more limited volume, defined by the contrast-enhanced T1 abnormality on postoperative MRI with a 2-cm CTV margin and a 3- to 5-mm PTV margin. The initial volume receives 46 Gy in 2 Gy daily fractions, and the boost volume receives 14 Gy in 2 Gy daily fractions (7). The landmark Stupp trial specified RT planning per EORTC recommendations (5).

Patterns of failure studies have shown that 80–90% of recurrences occur within 2–3 cm of the surgical cavity, which has guided the development of the conventional treatment volumes described above. The rationale behind CTV margins includes the belief that areas of T1 contrast enhancement represent the area of greatest tumor cell density and areas of T2 or FLAIR abnormality correspond to diffusely infiltrating disease (43). Various studies have evaluated the impact of smaller initial treatment volumes on patterns of failure and overall outcomes. The Department of Radiation Oncology at MD Anderson Cancer Center utilizes an alternative treatment volume specification that includes the resection cavity and any contrast enhancing residual disease with a 2-cm margin, while purposefully excluding any peritumoral edema, followed by a 5-mm PTV expansion, treated to 50 Gy in 2 Gy fractions, followed by an additional 10 Gy in five fractions to the resection cavity with a 5-mm PTV expansion. This is generally a smaller volume than the prescribed RTOG specifications. A study by Kumar et al. randomized patients to treatment volumes as per RTOG criteria (arm A) and the MD Anderson criteria (arm B). The PTV was significantly smaller in arm B (436 vs 246 cm^3^, *p* = 0.001) (44). The results of this trial (published in abstract form only), showed no difference in recurrence patterns, however, arm B did show an improved mean overall survival and improved quality of life outcomes. The null hypothesis was rejected and there was sufficient statistical evidence to suggest an increased overall survival for patients treated with smaller treatment volumes. A large retrospective review from Wake Forest reviewing RT outcomes with different CTV expansions showed no difference in treatment patterns of failure when using 5-, 10-, and 15- to 20-mm CTV margins, when using modern RT techniques with concurrent and adjuvant TMZ (45). The New Approaches to Brain Tumor Therapy consortium has used 5-mm margins in three different phase 2 studies, utilizing novel agents in addition to RT and TMZ, with significant improvement in survival over the chemoRT arm of the Stupp trial (5, 46). These favorable results may indicate that smaller CTV margins may not be inferior to the larger volumes used in the landmark EORTC trial. While further investigation is clearly warranted to more clearly characterize the optimal treatment volume margin, there is evidence that limited margins may not compromise recurrence patterns or survival, and may actually improve outcomes. One hypothesis regarding this is that more limited irradiated volumes may limit the lymphopenia, which has been associated with survival (43).

One drawback of conventional imaging is the uncertainty inherent in defining true tumor from edema. One workaround may include the use of novel positron emission tomographic (PET) tracers to facilitate improved RT planning in GBM. While ^18^fluorodeoxyglucose is the most commonly used radiolabeled tracer in PET scans, its use in neuroimaging is limited due to the high background activity in the brain, whereas radiolabeled amino acid tracers are preferentially absorbed by tumor cells due to excess amino acid transporters (47). The amino acid *O*-(2-[^18^F]-fluoroethyl)-l-tyrosine (FET) in conjunction with MRI was shown to improve the identification of cellular glioma tissue allowing for histologic diagnosis, with one study showing a sensitivity and specificity of 96 and 53% with MRI alone, and 93 and 94% with combined MRI and FET (48). Another amino acid tracer^11^ C methionine positron emission tomography (MET-PET) has been compared to gadolinium-enhanced T1-weighted MRI in the postoperative setting and has shown that the size and location of residual MET uptake varies considerably from postoperative MRI abnormality, which may aid in more accurate RT target delineation (49). A study from the same institution utilized MET-PET (SPECT)/CT/MRI imaging in patients with recurrent high-grade glioma undergoing stereotactic hypofractionated reirradiation in order to better delineate the GTV. The novel imaging arm showed improved median survival time compared with CT/MRI alone (9 vs 5 months, *p* = 0.03); however, the multivariate analysis suggests that the addition of temozolamide played a greater role than the use of PET/SPECT planning, thus given the nonrandomized nature of this trial and small number of patients, the main take home point of this study is the safety and feasibility of this approach (50). Furthermore, a study evaluating the role of dose escalation in GBM that utilized pretreatment MET-PET imaging demonstrated that patients whose treatment target volumes failed to include the region of increase MET-PET uptake had a higher risk of noncentral failure (*p* < 0.001), rejecting the null hypothesis, thus demonstrating a benefit when treatment volumes were delineated with the utilization of MET-PET to define areas at risk of recurrence not visible on MRI (51).

There may be a role for diffusion tensor imaging (DTI) to aid with RT target delineation. This MR-based technique is sensitive to subtle disruptions of white-matter (tensor) tracts, allowing for the identification of tumor cell infiltration along white-matter tracts in high-grade gliomas. This has been investigated in RT planning and may reduce treatment volume size, while simultaneously preserving coverage of likely routes of microscopic spread (52). The white-matter abnormalities seen on DTI that are normally invisible on conventional CT or MRI have been called image-based high-risk volumes (IHV) by Jena et al. This same study executed seven theoretical treatment plans utilizing a CTV generated by adding a 1-cm margin to the IHV_DTI_ compared to conventional treatment volumes. The use of DTI allowed for a mean reduction in the size of the PTV of 35% (range 18–46%) allowing for dose escalation (mean 67 Gy, range 64–74 Gy), yet with normal tissue complication probabilities that matched conventional treatment plans (53). This study demonstrates the value of DTI in shrinking RT volumes while preserving accuracy, paving the way for future studies with further dose escalation.

## Combined RT and Immunotherapy

Immune checkpoint blockade *via* the inhibition of cytotoxic T-lymphocyte associated protein 4 (CTLA-4) and programmed death protein 1 (PD-1) receptors has shown recent success in the treatment of melanoma, non-small cell lung cancer, and renal cell carcinoma. The benefit of RT has traditionally been attributed to its local, cytotoxic effect through DNA damage. Newer evidence has shown that RT increases immunogenic cell death by increasing antigen presentation and promoting a proinflammatory tumor microenvironment, enhancing antitumor T cell recruitment and the overall antitumor response by the host (54). This immunologic response to localized radiation may lead to tumor regression at distant non-irradiated tumor sites; a phenomenon termed the abscopal effect (55). As such, there has been enthusiasm to study the combination of immunotherapy and radiation whose synergy may stimulate a more robust antitumor response, contributing to tumor remission.

More recent evidence has shown that the central nervous system undergoes constant immune surveillance, aided by functional lymphatic vessels lining the dural sinuses (56). Similar to other malignancies, primary CNS tumors like GBM are able to evade the immune system through a number of immunosuppressive mechanisms, including the upregulation of immune checkpoints (57). In GBM, programmed death ligand-1 (PD-L1) has been demonstrated to be upregulated after the loss of phosphatase and tensin homolog (PTEN) and activation of the phosphatidylinositol-3-OH kinase (PI3K) pathway (58). This study showed that T-cells were less effective at lysing mutant PTEN, demonstrating PD-L1 expression as a primary immunosuppressive mechanism. Another study showed that the local inflammatory response induced by RT leads to upregulation of PD-L1 on cancer cells, macrophages, and dendritic cells, lending further support for the use of radiation with immune checkpoint blockade (59).

The combination of immunotherapy and radiation has shown efficacy in preclinical trials, including a study by Newcomb et al. testing the use of whole-brain RT (4 Gy in two fractions) and vaccination with irradiated GL261 cells secreting granulocyte-macrophage colony-stimulating factor, a form of active immunotherapy in GL261 murine gliomas (60). The experimental murine GL261 model used in this trial was intended to emulate the invasive, aggressive growth seen in human brain tumors. The delivery of whole-brain RT led to upregulation of MHC-I expression *in vitro*, which increased CD4+ and CD8+ T cell infiltration. The authors hypothesized that one mechanism by which glioma tumor cells evade immunosurveillance is by downregulating expression of MHC molecules at the edge of the tumor. The null hypothesis of the study design was an equal survival among mice receiving (a) no treatment, (b) WBRT in two fractions of 4 Gy, (c) vaccination with irradiated GL261 cells secreting granulocyte-macrophage colony-stimulating factor, or (d) WBRT and vaccination. The increased expression seen in the combined RT plus vaccination arm in this trial resulted in rejection of the null hypothesis, with results portending long-term mouse survival of 40–80%, compared to only 0–10% in the arm receiving no treatment, radiation alone, or vaccination alone (*p* < 0.002).

A more recent trial by Zeng et al. involved the implantation of mice with the GL261 murine glioma cell line followed by stratification into 1 of 4 arms including: no treatment, stereotactic radiation alone, anti-PD-1 antibody alone, and SRS plus anti-PD-1 antibody, with the null hypothesis of no difference in survival among treatment arms (61). RT, comprised of 10 Gy, was delivered in a single fraction centered on the tumor. The survival in the combination arm (53 days) was almost double that of the monotherapy arms (25, 28, and 27 days for the control group, radiation only group, and anti-PD-1 group, respectively), rejecting the null hypothesis (*p* < 0.05), and immunologic data showed significantly increased tumor infiltration by cytotoxic T cells (CD8+/interferon-γ+/tumor necrosis factor-α+) and decreased regulatory T cells (CD4+/FOXP3) in the combined arm. Long-term survival of over 180 days was seen in 15–40% of the mice. The results of this trial provided strong preclinical data to support the combination of immune checkpoint blockade with RT for patients with GBM.

Another study by Belcaid et al. examined the use of a triple therapy regimen for a murine glioma model involving a combination of CTLA-4 checkpoint blockade, SRS, and activation of 4-1BB agonist antibodies (a costimulatory signal expressed by T lymphocytes). RT was given as 10 Gy in a single fraction, similar to the previous study (62). The first null hypothesis studied was there being no difference between treatment with 4-1BB and no treatment and the results showed that treatment with 4-1BB agonist antibodies did not enhance survival when compared to untreated mice, nor did focal RT alone, failing to reject the null hypothesis. The second hypothesis of combination of therapy with CTLA-4 blockade and focal RT prolonged overall survival, rejecting the null hypothesis. Furthermore, the triple therapy regimen with SRS, 4-1BB activation, and CTLA-4 blockade extended the median survival from 24 days when treated with focal RT to 67 days (*p* < 0.05 vs all other treatment modalities) with 50% of mice as long-term survivors. Laboratory studies of the triple therapy regimen arm showed significantly higher numbers of infiltrating CD4+ T cells. These findings suggested that radiation is synergistic when combined with immunotherapy.

Checkmate 143 (NCT02017717) was a prospective trial evaluating the role of immunotherapy in recurrent GBM testing nivolumab, which is a fully human IgG4 monoclonal antibody inhibitor of the PD-1 receptor vs bevacizumab. Recent results show a median overall survival of 9.8 months with nivolumab and 10.0 months with bevacizumab, and a 12-month overall survival rate of 42% in both arms (63). Other clinical trials are currently ongoing in order to validate the efficacy of immune checkpoint blockade and RT in the up front setting. Checkmate 548 is a randomized phase 2 single blind study of TMZ with RT combined with nivolumab or placebo in newly diagnosed GBM with MGMT promoter methylation (NCT02667587); whereas Checkmate 498 is a phase 3 randomized study of nivolumab vs TMZ each in combination with RT for newly diagnosed GBM with an unmethylated MGMT promoter (NCT02617589). Another phase 2 trial is evaluating the combination of RT, TMZ, and pembrolizumab, another anti-PD-1 antibody, for newly diagnosed GBM (NCT02530502). Finally, for recurrent GBM, there is an ongoing phase 1 trial evaluating fractionated stereotactic RT in combination with bevacizumab and pembrolizumab (NCT02313272). If these trials show benefit, this will provide circumstantial evidence that radiation may increase the antigenicity of the tumor and thus render it more susceptible to immune-based therapies.

## Neural Stem Cells (NSCs) and GBM

The largest concentration of NSCs have been found in the subventricular zone (SVZ) located between the lateral ventricles and the striatal parenchyma (64). NSCs are essential in regulating and maintaining homeostasis in the brain and can migrate through parenchyma, similar to malignant gliomas. GBM is thought to consist of a self-renewing population of cancer stem cells (CSCs) that promote tumorigenesis and treatment resistance. A breakthrough was achieved in 2002 when the initial evidence for stem-cell like characteristics in human brain tumors was discovered through the isolation of clonogenic precursors from postoperative specimens of human GBM (65, 66). CSCs have been grown *in vitro* where they form neurospheres, are able to self-renew and have also been grown as *in vivo* xenografts, forming heterogeneous tumors morphologically identical to the original tumor (67). A mutual characteristic of NSCs and CSCs is that they may readily proliferate in culture when stimulated with the appropriate growth factors. NSCs differ from the bulk tumor population given their ability to show epigenetic changes and the role for microRNAs to regulate gene expression (68, 69).

In addition to their similarities in maintaining homeostasis and promoting growth, GBM CSCs and NSCs have been shown to have similar gene-expression profiles, lending support to the concept that CSCs are malignant derivatives of NSCs. Several of the signaling pathways shared between NSCs and CSCs involved in neural developments include PTEN, Notch, Sonic hedgehog, telomerase, Wnt, and transforming growth factor-β (68, 70). The shared signaling pathways and gene-expression profiles seen in both NSCs and CSCs suggest that GBM CSCs may be derivatives of malignantly transformed NSCs, or perhaps they may originate from mature cells that dedifferentiated and gained self-renewal abilities (71).

Given the putative role of SVZ stem cells in gliomagenesis, SVZ targeting has been proposed as a potential target for radiation therapy (72). Lim et al. proposed therapy planning that considered the locations of the contrast-enhancing GBM in relation to the SVZ. Tumors were placed into four different groups: contact with SVZ and cortical infiltration (group I); contact with SVZ without cortical infiltration (group II); cortical infiltration without SVZ contact (group III); no SVZ contact or cortical infiltration (group IV) (73). Further studies have also grouped GBM spatial distribution patterns at initial diagnosis using the Lim criteria and found that roughly a quarter of patients fall into each group (74).

Furthermore, tumors contacting the SVZ may be more invasive with a higher potential to recruit migratory progenitor cells. Disease outcomes based on the Lim criteria have showed that when comparing group I tumors with groups II–IV, only 39% of patients with type I tumors were recurrence free and alive at 6 months, which was significantly less compared with all other groups (67%; *p* = 0.01). The authors concluded that GBM involvement of the SVZ, regardless of cortical involvement led to quicker progression and decreased survival (74). Similarly, other studies have also shown improved progression-free and overall survival in patients with tumors without SVZ contact, whereas SVZ proximity (<10 mm) and infiltration was seen in patients with short-term survival (75–77).

Proximity to the SVZ may also impact patterns of recurrence. Patients with group I GBMs were most likely to have multifocal disease at diagnosis compared to groups II–IV. Furthermore, group I GBMs are postulated to be most associated with SVZ stem cells as these tumors are spatially located near the SVZ, and most likely to recur distally in the brain, while group IV tumors are more likely to recur within 2 cm of the original disease margin (73, 78, 79). This potential difference in recurrence pattern has the potential to impact local treatment strategies.

Given that the available evidence suggests GBM proximity to the SVZ impacts recurrence patterns and prognosis, it is possible that irradiation of the CSC niche in the SVZ may provide therapeutic benefit in selected patients (80). Eight retrospective studies and one prospective study have been published evaluating the impact of incidental inclusion of the SVZ in radiation treatment plans; results have been mixed (80–88). One the more provocative results was seen in a prospective phase 2 trial by Luchi et al., which reported outcomes of 46 newly diagnosed GBM patients treated with hypofractionated IMRT, with the ipsilateral SVZ receiving an incidental dose of 50–60 Gy. The presence of SVZ necrosis was found to be the only variable significantly associated with prolonged overall survival on multivariate analysis (36.2 vs 13.3 months, HR 4.08; CI 1.97–9.10, *p* = 0.0001) (83) This large survival difference on the basis of SVZ necrosis is encouraging; however, these results are from a small, single-institution, nonrandomized study and may reflect the fact that these patients live long enough to develop necrosis, as the author’s report the median time to SVZ necrosis of 16.1 months. A separate study also showed an improved overall survival with increasing mean dose to the ipsilateral SVZ, yet patients who received a higher dose to the contralateral SVZ demonstrated worse progression-free survival; this may have reflected patients with more diffuse tumors that crossed midline thereby leading to larger SVZ doses and worse outcomes (89).

These studies have significant limitations. The retrospective nature of them and heterogeneous patient population make the role of SVZ irradiation difficult to decipher. Moreover, the technique implies that the CSC pool derives from the SVZ population. This is at best speculative given evidence that more differentiated tumor cells can exist in equilibrium with more stem-like phenotypes (90); such ability might explain tumor recurrence after treatment independent of any SVZ CSC pool. In addition, it is unclear what volume and dose of irradiation would sufficiently treat the SVZ.

A prospective trial that treated 30 GBM patients with standard 60 Gy RT and TMZ attempted to determine if limiting doses to neural progenitor cell (NPC) regions with IMRT would improve neurocognitive outcomes (NCT01478854). The NPC regions were defined as the SVZ (0.5 cm adjacent to lateral wall of lateral ventricle) and hippocampus (expanded 5 mm). The results, published in abstract form, show that while two patients did recur in the spared NPC regions, higher mean RT dose to the bilateral SVZ was associated with worsening cognitive performance and there was no association between SVZ dose and disease outcomes (91).

There is one ongoing phase 2 randomized trial testing treatment of GBM with standard RT and TMZ +/− deliberate irradiation of the ipsilateral SVZ to 60 Gy and contralateral SVZ to 46 Gy (NCT02177578). It is our view that SVZ irradiation is unlikely to be of significant benefit given the biology of GBM CSCs; however, this prospective trial will provide important data regarding its role. Another important consideration as well is the potential harm in treating larger volumes known to carry risk of substantial late effects, including neurocognitive toxicity and radiation necrosis (92).

## Summary

The past few decades have seen enormous leaps in the ability of modern RT to deliver highly conformal radiation doses to maximize treatment efficacy while limiting normal tissue toxicity. The addition of concurrent and adjuvant TMZ has demonstrated an improvement in overall survival in patients, and the results of trials evaluating hypofractionation with concomitant TMZ have allowed for aggressive treatment in the elderly population without a detriment on quality of life or deterioration in treatment compliance. Contemporary treatment is moving toward more precise target delineation consisting of smaller, more accurate volumes, aided by advances in neuroimaging and radiolabeled amino acids. Additionally, the role of radiation in priming the immune system to help control GBM is in its nascent stages. Finally, there is evidence that specific targeting of stem cell niches by deliberate inclusion of the SVZ in the radiation field may portend a better prognosis in a select group of patients, with further studies underway to better elucidate this area of uncertainty. Continuing improvements in understanding of this aggressive and devastating disease will allow us to better refine treatment in the future to further improve and extend the lives of our patients.

## Author Contributions

RR is the corresponding author. JM, AGW, RM are all equal contributors.

## Conflict of Interest Statement

The authors declare that the research was conducted in the absence of any commercial or financial relationships that could be construed as a potential conflict of interest.

## References

[1] Brandes AA. State-of-the-art treatment of high-grade brain tumors. Semin Oncol (2003) 30:4–9. 10.1053/j.seminoncol.2003.11.028 14765377

[2] Walker MD Green SB Byar DP Alexander E Jr Batzdorf U Brooks WH Randomized comparisons of radiotherapy and nitrosoureas for the treatment of malignant glioma after surgery. N Engl J Med (1980) 303:1323–9. 10.1056/NEJM198012043032303 7001230

[3] Walker MD Alexander E Jr Hunt WE MacCarty CS Mahaley MS Jr Mealey J Jr Evaluation of BCNU and/or radiotherapy in the treatment of anaplastic gliomas. A cooperative clinical trial. J Neurosurg (1978) 49:333–43. 10.3171/jns.1978.49.3.0333 355604

[4] Walker MD Strike TA Sheline GE An analysis of dose-effect relationship in the radiotherapy of malignant gliomas. Int J Radiat Oncol Biol Phys (1979) 5:1725–31. 10.1016/0360-3016(79)90553-4 231022

[5] Stupp R Mason WP van den Bent MJ Weller M Fisher B Taphoorn MJ Radiotherapy plus concomitant and adjuvant temozolomide for glioblastoma. N Engl J Med (2005) 352:987–96. 10.1056/NEJMoa043330 15758009

[6] Chinot OL Wick W Mason W Henriksson R Saran F Nishikawa R Bevacizumab plus radiotherapy-temozolomide for newly diagnosed glioblastoma. N Engl J Med (2014) 370:709–22. 10.1056/NEJMoa1308345 24552318

[7] Gilbert MR Dignam JJ Armstrong TS Wefel JS Blumenthal DT Vogelbaum MA A randomized trial of bevacizumab for newly diagnosed glioblastoma. N Engl J Med (2014) 370:699–708. 10.1056/NEJMoa1308573 24552317 PMC4201043

[8] Babu R Komisarow JM Agarwal VJ Rahimpour S Iyer A Britt D Glioblastoma in the elderly: the effect of aggressive and modern therapies on survival. J Neurosurg (2016) 124:998–1007. 10.3171/2015.4.JNS142200 26452121

[9] Curran WJ Jr Scott CB Horton J Nelson JS Weinstein AS Fischbach AJ Recursive partitioning analysis of prognostic factors in three radiation therapy oncology group malignant glioma trials. J Natl Cancer Inst (1993) 85:704–10. 10.1093/jnci/85.9.704 8478956

[10] Ramakrishna R Kim L Rostomily R Glioblastoma: the importance of not being ageist. World Neurosurg (2011) 76(5):369–70. 10.1016/j.wneu.2011.08.028 22152553

[11] Scott JG Suh JH Elson P Barnett GH Vogelbaum MA Peereboom DM Aggressive treatment is appropriate for glioblastoma multiforme patients 70 years old or older: a retrospective review of 206 cases. Neuro Oncol (2011) 13:428–36. 10.1093/neuonc/nor005 21363881 PMC3064699

[12] Keime-Guibert F Chinot O Taillandier L Cartalat-Carel S Frenay M Kantor G Radiotherapy for glioblastoma in the elderly. N Engl J Med (2007) 356:1527–35. 10.1056/NEJMoa065901 17429084

[13] Roa W Brasher PM Bauman G Anthes M Bruera E Chan A Abbreviated course of radiation therapy in older patients with glioblastoma multiforme: a prospective randomized clinical trial. J Clin Oncol (2004) 22:1583–8. 10.1200/JCO.2004.06.082 15051755

[14] Perry JR Laperriere N O’Callaghan CJ Brandes AA Menten J Phillips C Short-course radiation plus temozolomide in elderly patients with glioblastoma. N Engl J Med (2017) 376:1027–37. 10.1056/NEJMoa1611977 28296618

[15] Wick W Platten M Meisner C Felsberg J Tabatabai G Simon M Temozolomide chemotherapy alone versus radiotherapy alone for malignant astrocytoma in the elderly: the NOA-08 randomised, phase 3 trial. Lancet Oncol (2012) 13:707–15. 10.1016/S1470-2045(12)70164-X 22578793

[16] Gilbert MR Wang M Aldape KD Stupp R Hegi ME Jaeckle KA Dose-dense temozolomide for newly diagnosed glioblastoma: a randomized phase III clinical trial. J Clin Oncol (2013) 31:4085–91. 10.1200/JCO.2013.49.6968 24101040 PMC3816958

[17] Malmstrom A Gronberg BH Marosi C Stupp R Frappaz D Schultz H Temozolomide versus standard 6-week radiotherapy versus hypofractionated radiotherapy in patients older than 60 years with glioblastoma: the Nordic randomised, phase 3 trial. Lancet Oncol (2012) 13:916–26. 10.1016/S1470-2045(12)70265-6 22877848

[18] Roa W Kepka L Kumar N Sinaika V Matiello J Lomidze D International atomic energy agency randomized phase III study of radiation therapy in elderly and/or frail patients with newly diagnosed glioblastoma multiforme. J Clin Oncol (2015) 33:4145–50. 10.1200/JCO.2015.62.6606 26392096

[19] Cabrera AR Kirkpatrick JP Fiveash JB Shih HA Koay EJ Lutz S Radiation therapy for glioblastoma: executive summary of an American Society for Radiation Oncology Evidence-Based Clinical Practice Guideline. Pract Radiat Oncol (2016) 6:217–25. 10.1016/j.prro.2016.03.007 27211230

[20] Carson KA Grossman SA Fisher JD Shaw EG. Prognostic factors for survival in adult patients with recurrent glioma enrolled onto the new approaches to brain tumor therapy CNS consortium phase I and II clinical trials. J Clin Oncol (2007) 25:2601–6. 10.1200/JCO.2006.08.1661 17577040 PMC4118746

[21] Wong ET Gautam S Malchow C Lun M Pan E Brem S. Bevacizumab for recurrent glioblastoma multiforme: a meta-analysis. J Natl Compr Canc Netw (2011) 9:403–7. 10.6004/jnccn.2011.0037 21464145

[22] Friedman HS Prados MD Wen PY Mikkelsen T Schiff D Abrey LE Bevacizumab alone and in combination with irinotecan in recurrent glioblastoma. J Clin Oncol (2009) 27:4733–40. 10.1200/JCO.2008.19.8721 19720927

[23] Verhoeff JJ Lavini C van Linde ME Stalpers LJ Majoie CB Reijneveld JC Bevacizumab and dose-intense temozolomide in recurrent high-grade glioma. Ann Oncol (2010) 21:1723–7. 10.1093/annonc/mdp591 20064829

[24] Gilbert MR Pugh SL Aldape K Sorensen AG Mikkelsen T Penas-Prado M NRG oncology RTOG 0625: a randomized phase II trial of bevacizumab with either irinotecan or dose-dense temozolomide in recurrent glioblastoma. J Neurooncol (2016) 131(1):193–9. 10.1007/s11060-016-2288-5 27770279 PMC5263144

[25] Reardon DA Desjardins A Vredenburgh JJ Gururangan S Sampson JH Sathornsumetee S Metronomic chemotherapy with daily, oral etoposide plus bevacizumab for recurrent malignant glioma: a phase II study. Br J Cancer (2009) 101:1986–94. 10.1038/sj.bjc.6605412 19920819 PMC2795427

[26] Sathornsumetee S Desjardins A Vredenburgh JJ McLendon RE Marcello J Herndon JE Phase II trial of bevacizumab and erlotinib in patients with recurrent malignant glioma. Neuro Oncol (2010) 12:1300–10. 10.1093/neuonc/noq099 20716591 PMC3018944

[27] Quant EC Norden AD Drappatz J Muzikansky A Doherty L Lafrankie D Role of a second chemotherapy in recurrent malignant glioma patients who progress on bevacizumab. Neuro Oncol (2009) 11:550–5. 10.1215/15228517-2009-006 19332770 PMC2765344

[28] Nieder C Astner ST Mehta MP Grosu AL Molls M. Improvement, clinical course, and quality of life after palliative radiotherapy for recurrent glioblastoma. Am J Clin Oncol (2008) 31:300–5. 10.1097/COC.0b013e31815e3fdc 18525311

[29] Combs SE Thilmann C Edler L Debus J Schulz-Ertner D. Efficacy of fractionated stereotactic reirradiation in recurrent gliomas: long-term results in 172 patients treated in a single institution. J Clin Oncol (2005) 23:8863–9. 10.1200/JCO.2005.03.4157 16314646

[30] Fogh SE Andrews DW Glass J Curran W Glass C Champ C Hypofractionated stereotactic radiation therapy: an effective therapy for recurrent high-grade gliomas. J Clin Oncol (2010) 28:3048–53. 10.1200/JCO.2009.25.6941 20479391 PMC2982785

[31] Vordermark D Kolbl O Ruprecht K Vince GH Bratengeier K Flentje M. Hypofractionated stereotactic re-irradiation: treatment option in recurrent malignant glioma. BMC Cancer (2005) 5:55. 10.1186/1471-2407-5-55 15924621 PMC1156875

[32] Shaw E Scott C Souhami L Dinapoli R Kline R Loeffler J Single dose radiosurgical treatment of recurrent previously irradiated primary brain tumors and brain metastases: final report of RTOG protocol 90-05. Int J Radiat Oncol Biol Phys (2000) 47:291–8. 10.1016/S0360-3016(99)00507-6 10802351

[33] Hall WA Djalilian HR Sperduto PW Cho KH Gerbi BJ Gibbons JP Stereotactic radiosurgery for recurrent malignant gliomas. J Clin Oncol (1995) 13:1642–8. 10.1200/JCO.1995.13.7.1642 7602353

[34] Shrieve DC Alexander E III Wen PY Fine HA Kooy HM Black PM Comparison of stereotactic radiosurgery and brachytherapy in the treatment of recurrent glioblastoma multiforme. Neurosurgery (1995) 36:275–82; discussion 282–4. 10.1097/00006123-199502000-00006 7731507

[35] Cho KH Hall WA Gerbi BJ Higgins PD McGuire WA Clark HB. Single dose versus fractionated stereotactic radiotherapy for recurrent high-grade gliomas. Int J Radiat Oncol Biol Phys (1999) 45:1133–41. 10.1016/S0360-3016(99)00336-3 10613305

[36] Combs SE Widmer V Thilmann C Hof H Debus J Schulz-Ertner D Stereotactic radiosurgery (SRS): treatment option for recurrent glioblastoma multiforme (GBM). Cancer (2005) 104:2168–73. 10.1002/cncr.21429 16220556

[37] Gorski DH Beckett MA Jaskowiak NT Calvin DP Mauceri HJ Salloum RM Blockage of the vascular endothelial growth factor stress response increases the antitumor effects of ionizing radiation. Cancer Res (1999) 59:3374–8. 10416597

[38] Zhuang HQ Yuan ZY Wang P. Research progress on the mechanisms of combined bevacizumab and radiotherapy. Recent Pat Anticancer Drug Discov (2014) 9:129–34. 10.2174/15748928113089990044 24032532

[39] Levin VA Bidaut L Hou P Kumar AJ Wefel JS Bekele BN Randomized double-blind placebo-controlled trial of bevacizumab therapy for radiation necrosis of the central nervous system. Int J Radiat Oncol Biol Phys (2011) 79:1487–95. 10.1016/j.ijrobp.2009.12.061 20399573 PMC2908725

[40] Gutin PH Iwamoto FM Beal K Mohile NA Karimi S Hou BL Safety and efficacy of bevacizumab with hypofractionated stereotactic irradiation for recurrent malignant gliomas. Int J Radiat Oncol Biol Phys (2009) 75:156–63. 10.1016/j.ijrobp.2008.10.043 19167838 PMC3659401

[41] Niyazi M Ganswindt U Schwarz SB Kreth FW Tonn JC Geisler J Irradiation and bevacizumab in high-grade glioma retreatment settings. Int J Radiat Oncol Biol Phys (2012) 82:67–76. 10.1016/j.ijrobp.2010.09.002 21030162

[42] Niyazi M Brada M Chalmers AJ Combs SE Erridge SC Fiorentino A ESTRO-ACROP guideline “target delineation of glioblastomas”. Radiother Oncol (2016) 118:35–42. 10.1016/j.radonc.2015.12.003 26777122

[43] Wernicke AG Smith AW Taube S Mehta MP. Glioblastoma: radiation treatment margins, how small is large enough? Pract Radiat Oncol (2016) 6:298–305. 10.1016/j.prro.2015.12.002 26952812

[44] Narendra Kumar RK Sharma SC Mukherjee KK Ritesh Kumar NK Gupta PK Bansal A RT-09 to compare the treatment outcomes of two different target volume delineation guidelines (RTOG vs MD Anderson) in glioblastoma multiforme patients: a prospective randomized study. Neuro Oncol (2012) 14(Suppl 6):vi133–41. 10.1093/neuonc/nos238

[45] Paulsson AK McMullen KP Peiffer AM Hinson WH Kearns WT Johnson AJ Limited margins using modern radiotherapy techniques does not increase marginal failure rate of glioblastoma. Am J Clin Oncol (2014) 37:177–81. 10.1097/COC.0b013e318271ae03 23211224 PMC4485493

[46] Grossman SA Ye X Piantadosi S Desideri S Nabors LB Rosenfeld M Survival of patients with newly diagnosed glioblastoma treated with radiation and temozolomide in research studies in the United States. Clin Cancer Res (2010) 16:2443–9. 10.1158/1078-0432.CCR-09-3106 20371685 PMC2861898

[47] Gzell C Back M Wheeler H Bailey D Foote M. Radiotherapy in glioblastoma: the past, the present and the future. Clin Oncol (R Coll Radiol) (2017) 29:15–25. 10.1016/j.clon.2016.09.015 27743773

[48] Pauleit D Floeth F Hamacher K Riemenschneider MJ Reifenberger G Muller HW O-(2-[18F]fluoroethyl)-L-tyrosine PET combined with MRI improves the diagnostic assessment of cerebral gliomas. Brain (2005) 128:678–87. 10.1093/brain/awh399 15689365

[49] Grosu AL Weber WA Riedel E Jeremic B Nieder C Franz M l-(methyl-11C) methionine positron emission tomography for target delineation in resected high-grade gliomas before radiotherapy. Int J Radiat Oncol Biol Phys (2005) 63:64–74. 10.1016/j.ijrobp.2005.01.045 16111573

[50] Grosu AL Weber WA Franz M Stark S Piert M Thamm R Reirradiation of recurrent high-grade gliomas using amino acid PET (SPECT)/CT/MRI image fusion to determine gross tumor volume for stereotactic fractionated radiotherapy. Int J Radiat Oncol Biol Phys (2005) 63:511–9. 10.1016/j.ijrobp.2005.01.056 16168843

[51] Tsien CI Brown D Normolle D Schipper M Piert M Junck L Concurrent temozolomide and dose-escalated intensity-modulated radiation therapy in newly diagnosed glioblastoma. Clin Cancer Res (2012) 18:273–9. 10.1158/1078-0432.CCR-11-2073 22065084 PMC3266840

[52] Berberat J McNamara J Remonda L Bodis S Rogers S. Diffusion tensor imaging for target volume definition in glioblastoma multiforme. Strahlenther Onkol (2014) 190:939–43. 10.1007/s00066-014-0676-3 24823897

[53] Jena R Price SJ Baker C Jefferies SJ Pickard JD Gillard JH Diffusion tensor imaging: possible implications for radiotherapy treatment planning of patients with high-grade glioma. Clin Oncol (R Coll Radiol) (2005) 17:581–90. 10.1016/j.clon.2005.04.012 16372482

[54] Demaria S Formenti SC. Sensors of ionizing radiation effects on the immunological microenvironment of cancer. Int J Radiat Biol (2007) 83:819–25. 10.1080/09553000701481816 17852561

[55] Formenti SC Demaria S. Systemic effects of local radiotherapy. Lancet Oncol (2009) 10:718–26. 10.1016/S1470-2045(09)70082-8 19573801 PMC2782943

[56] Louveau A Smirnov I Keyes TJ Eccles JD Rouhani SJ Peske JD Structural and functional features of central nervous system lymphatic vessels. Nature (2015) 523:337–41. 10.1038/nature14432 26030524 PMC4506234

[57] D’Souza NM Fang P Logan J Yang J Jiang W Li J. Combining radiation therapy with immune checkpoint blockade for central nervous system malignancies. Front Oncol (2016) 6:212. 10.3389/fonc.2016.00212 27774435 PMC5053992

[58] Parsa AT Waldron JS Panner A Crane CA Parney IF Barry JJ Loss of tumor suppressor PTEN function increases B7-H1 expression and immunoresistance in glioma. Nat Med (2007) 13:84–8. 10.1038/nm1517 17159987

[59] Deng L Liang H Burnette B Weicheslbaum RR Fu YX. Radiation and anti-PD-L1 antibody combinatorial therapy induces T cell-mediated depletion of myeloid-derived suppressor cells and tumor regression. Oncoimmunology (2014) 3:e28499. 10.4161/onci.28499 25050217 PMC4063144

[60] Newcomb EW Demaria S Lukyanov Y Shao Y Schnee T Kawashima N The combination of ionizing radiation and peripheral vaccination produces long-term survival of mice bearing established invasive GL261 gliomas. Clin Cancer Res (2006) 12:4730–7. 10.1158/1078-0432.CCR-06-0593 16899624

[61] Zeng J See AP Phallen J Jackson CM Belcaid Z Ruzevick J Anti-PD-1 blockade and stereotactic radiation produce long-term survival in mice with intracranial gliomas. Int J Radiat Oncol Biol Phys (2013) 86:343–9. 10.1016/j.ijrobp.2012.12.025 23462419 PMC3963403

[62] Belcaid Z Phallen JA Zeng J See AP Mathios D Gottschalk C Focal radiation therapy combined with 4-1BB activation and CTLA-4 blockade yields long-term survival and a protective antigen-specific memory response in a murine glioma model. PLoS One (2014) 9:e101764. 10.1371/journal.pone.0101764 25013914 PMC4094423

[63] Reardon DA Omuro A Brandes AA Rieger J Wick A Sepulveda J OS10.3 randomized phase 3 study evaluating the efficacy and safety of nivolumab vs bevacizumab in patients with recurrent glioblastoma: CheckMate 143. Neuro Oncol (2017) 19:21 10.1093/neuonc/nox036.071

[64] Sanai N Alvarez-Buylla A Berger MS Neural stem cells and the origin of gliomas. N Engl J Med (2005) 353:811–22. 10.1056/NEJMra043666 16120861

[65] Ignatova TN Kukekov VG Laywell ED Suslov ON Vrionis FD Steindler DA. Human cortical glial tumors contain neural stem-like cells expressing astroglial and neuronal markers in vitro. Glia (2002) 39:193–206. 10.1002/glia.10094 12203386

[66] Galli R Binda E Orfanelli U Cipelletti B Gritti A De Vitis S Isolation and characterization of tumorigenic, stem-like neural precursors from human glioblastoma. Cancer Res (2004) 64:7011–21. 10.1158/0008-5472.CAN-04-1364 15466194

[67] Singh SK Hawkins C Clarke ID Squire JA Bayani J Hide T Identification of human brain tumour initiating cells. Nature (2004) 432:396–401. 10.1038/nature03128 15549107

[68] Silber J Lim DA Petritsch C Persson AI Maunakea AK Yu M miR-124 and miR-137 inhibit proliferation of glioblastoma multiforme cells and induce differentiation of brain tumor stem cells. BMC Med (2008) 6:14. 10.1186/1741-7015-6-14 18577219 PMC2443372

[69] Vescovi AL Galli R Reynolds BA. Brain tumour stem cells. Nat Rev Cancer (2006) 6:425–36. 10.1038/nrc1889 16723989

[70] Godlewski J Nowicki MO Bronisz A Williams S Otsuki A Nuovo G Targeting of the Bmi-1 oncogene/stem cell renewal factor by microRNA-128 inhibits glioma proliferation and self-renewal. Cancer Res (2008) 68:9125–30. 10.1158/0008-5472.CAN-08-2629 19010882

[71] Sundar SJ Hsieh JK Manjila S Lathia JD Sloan A. The role of cancer stem cells in glioblastoma. Neurosurg Focus (2014) 37:E6. 10.3171/2014.9.FOCUS14494 25434391

[72] Smith AW Mehta MP Wernicke AG. Neural stem cells, the subventricular zone and radiotherapy: implications for treating glioblastoma. J Neurooncol (2016) 128:207–16. 10.1007/s11060-016-2123-z 27108274

[73] Lim DA Cha S Mayo MC Chen MH Keles E VandenBerg S Relationship of glioblastoma multiforme to neural stem cell regions predicts invasive and multifocal tumor phenotype. Neuro Oncol (2007) 9:424–9. 10.1215/15228517-2007-023 17622647 PMC1994099

[74] Jafri NF Clarke JL Weinberg V Barani IJ Cha S. Relationship of glioblastoma multiforme to the subventricular zone is associated with survival. Neuro Oncol (2013) 15:91–6. 10.1093/neuonc/nos268 23095230 PMC3534420

[75] Sonoda Y Saito R Kanamori M Kumabe T Uenohara H Tominaga T. The association of subventricular zone involvement at recurrence with survival after repeat surgery in patients with recurrent glioblastoma. Neurol Med Chir (Tokyo) (2014) 54:302–9. 10.2176/nmc.oa.2013-0226 24390189 PMC4533477

[76] Adeberg S Bostel T Konig L Welzel T Debus J Combs SE. A comparison of long-term survivors and short-term survivors with glioblastoma, subventricular zone involvement: a predictive factor for survival? Radiat Oncol (2014) 9:95. 10.1186/1748-717X-9-95 24758192 PMC4011838

[77] Young GS Macklin EA Setayesh K Lawson JD Wen PY Norden AD Longitudinal MRI evidence for decreased survival among periventricular glioblastoma. J Neurooncol (2011) 104:261–9. 10.1007/s11060-010-0477-1 21132516 PMC3151407

[78] Adeberg S Konig L Bostel T Harrabi S Welzel T Debus J Glioblastoma recurrence patterns after radiation therapy with regard to the subventricular zone. Int J Radiat Oncol Biol Phys (2014) 90:886–93. 10.1016/j.ijrobp.2014.07.027 25220720

[79] Chen L Chaichana KL Kleinberg L Ye X Quinones-Hinojosa A Redmond K. Glioblastoma recurrence patterns near neural stem cell regions. Radiother Oncol (2015) 116:294–300. 10.1016/j.radonc.2015.07.032 26276527 PMC4857206

[80] Gupta T Nair V Paul SN Kannan S Moiyadi A Epari S Can irradiation of potential cancer stem-cell niche in the subventricular zone influence survival in patients with newly diagnosed glioblastoma? J Neurooncol (2012) 109:195–203. 10.1007/s11060-012-0887-3 22555992

[81] Evers P Lee PP DeMarco J Agazaryan N Sayre JW Selch M Irradiation of the potential cancer stem cell niches in the adult brain improves progression-free survival of patients with malignant glioma. BMC Cancer (2010) 10:384. 10.1186/1471-2407-10-384 20663133 PMC2918578

[82] Lee P Eppinga W Lagerwaard F Cloughesy T Slotman B Nghiemphu PL Evaluation of high ipsilateral subventricular zone radiation therapy dose in glioblastoma: a pooled analysis. Int J Radiat Oncol Biol Phys (2013) 86:609–15. 10.1016/j.ijrobp.2013.01.009 23462418

[83] Iuchi T Hatano K Kodama T Sakaida T Yokoi S Kawasaki K Phase 2 trial of hypofractionated high-dose intensity modulated radiation therapy with concurrent and adjuvant temozolomide for newly diagnosed glioblastoma. Int J Radiat Oncol Biol Phys (2014) 88:793–800. 10.1016/j.ijrobp.2013.12.011 24495592

[84] Chen L Guerrero-Cazares H Ye X Ford E McNutt T Kleinberg L Increased subventricular zone radiation dose correlates with survival in glioblastoma patients after gross total resection. Int J Radiat Oncol Biol Phys (2013) 86:616–22. 10.1016/j.ijrobp.2013.02.014 23540348 PMC3996451

[85] Ravind R Prameela C Dinesh M P0111 sub-ventricular zone irradiation in glioblastoma: can it increase survival? Eur J Cancer (2015) 51:e23 10.1016/j.ejca.2015.06.069

[86] Slotman B Eppinga W de Haan P Lagerwaard F Is irradiation of potential cancer stem cell niches in the subventricular zones indicated in GBM? Int J Radiat Oncol Biol Phys (2011) 81:S184 10.1016/j.ijrobp.2011.06.328

[87] Elicin O Inac E Uzel EK Karacam S Uzel OE. Relationship between survival and increased radiation dose to subventricular zone in glioblastoma is controversial. J Neurooncol (2014) 118:413–9. 10.1007/s11060-014-1424-3 24668610

[88] Chua M Kusumawidjaja G Gan P Tan DH Chua ET Tham CK Dose-escalated intensity modulated radiotherapy (IMRT) and increased radiation doses to subventricular zones (SVZ) in treatment outcomes of patients with glioblastoma multiforme (GBM). Am Soc Radiat Oncol (2014) 90:S289–90. 10.1200/jco.2014.32.15_suppl.e13031

[89] Gupta T Nair V Jalali R. Stem cell niche irradiation in glioblastoma: providing a ray of hope? CNS Oncol (2014) 3:367–76. 10.2217/cns.14.39 25363009 PMC6113729

[90] Safa AR Saadatzadeh MR Cohen-Gadol AA Pollok KE Bijangi-Vishehsaraei K. Glioblastoma stem cells (GSCs) epigenetic plasticity and interconversion between differentiated non-GSCs and GSCs. Genes Dis (2015) 2:152–63. 10.1016/j.gendis.2015.02.001 26137500 PMC4484766

[91] Redmond K Ye X Assadi R McIntyre R Moore J Ford E Rtrb-17 neural progenitor cell (Npc) sparing radiation therapy plus temozolomide in newly diagnosed glioblastoma (Gbm) associated with cognitive function but not tumor outcomes: results of a single arm prospective clinical trial. Neuro Oncol (2015) 17:v198–9. 10.1093/neuonc/nov231.17

[92] Chang EL Wefel JS Hess KR Allen PK Lang FF Kornguth DG Neurocognition in patients with brain metastases treated with radiosurgery or radiosurgery plus whole-brain irradiation: a randomised controlled trial. Lancet Oncol (2009) 10:1037–44. 10.1016/S1470-2045(09)70263-3 19801201

